# Prevalence and Determinants of Frozen Shoulder in Patients with Diabetes: A Single Center Experience from Pakistan

**DOI:** 10.7759/cureus.1544

**Published:** 2017-08-06

**Authors:** Faisal Inayat, Nouman Safdar Ali, Haroon Shahid, Fariha Younus

**Affiliations:** 1 Department of Medicine, New York-Presbyterian Hospital, Weill Cornell Medical College, New York City, NY, USA; 2 Department of Medicine, Jinnah Hospital, Allama Iqbal Medical College, Lahore, Pakistan; 3 Department of Radiology, Shaukat Khanum Memorial Cancer Hospital & Research Centre, Lahore, Pakistan; 4 Department of Medicine Unit 4, Services Hospital, Services Institute of Medical Sciences, Lahore, Pakistan

**Keywords:** prevalence, determinants, frozen shoulder, diabetes mellitus, awareness, public health

## Abstract

Introduction

Frozen shoulder (FS) or adhesive capsulitis is a constellation of symptoms like pain, stiffness, and/or functional deficit at the glenohumeral joint. It is one of the musculoskeletal complications in patients with diabetes that can be particularly debilitating. The aim of this study is to estimate the prevalence of FS and to compare the determinants of this disease in a population with diabetes from Lahore, Pakistan.

Materials and Methods

We carried out this cross-sectional study on a systematically randomized sample of 80 patients with diabetes. It included 38 males and 42 females from 2,964 patients registered at the Diabetes Management Center, Services Hospital Lahore, Pakistan. The study was conducted in the months of April, May, and June 2017. A structured questionnaire was designed and the responses of patients were recorded at the clinic after informed verbal and written consent. The questionnaire outlined the key factors that can lead to a higher frequency of FS in patients with diabetes.

Results

Thirty-three of the total 80 respondents included in the study were diagnosed with FS. The estimated prevalence of FS in diabetics from this data was 41.3% in Lahore, which is an urban area of Pakistan with a population of more than seven million. Female sex, insulin dependence, uncontrolled blood glucose levels, and a positive family history were associated with a significantly higher prevalence of FS. In our study, most patients with FS were in Stage 1 of the disease and had unilateral involvement.

Conclusion

The present study shows that the prevalence of FS is higher in patients with diabetes residing in Lahore than in comparable foreign populations with diabetes. It can be attributed to socioeconomic status, lack of awareness, a higher threshold for diagnosis, and/or poor glycemic control. Mass awareness campaigns, especially for female patients with diabetes, are required to be initiated to create awareness about the disease and to facilitate early diagnosis and appropriate management. In-depth and multicenter studies are needed to further explore the association between FS and diabetes.

## Introduction

Frozen shoulder (FS) or adhesive capsulitis or periarthritis is characterized by the gradual development of restricted motion at the shoulder joint with nonspecific radiographic findings. Patients generally complain of severe shoulder pain with an inability to sleep on the affected side as the disease progresses. There are three clinical stages of frozen shoulder: freezing stage, frozen stage, and thawing stage. The freezing stage usually lasts from two to nine months with moderate to severe shoulder pain and stiffness. In the frozen stage, stiffness increases. This stage lasts for an average of 4 to 14 months and the component of pain is reduced. The thawing stage continues for about 5 to 24 months and constitutes gradual relief from the symptoms and recovery of range of motion.

FS mainly affects the older population, with a female predominance. The precise prevalence of FS is unknown, but authors have quoted figures of 2%–5% in the general population [1]. Those with prolonged shoulder immobility (minor upper limb trauma, overuse injury, surgery, and/or neurosurgery) or systemic diseases (diabetes, thyroid disorders, osteoporosis, Dupuytren’s contracture, cardiovascular disease, and stroke) are at a higher risk [2-10]. Parkinson’s syndrome and human immunodeficiency virus (HIV) has also been rarely associated with FS [11-13].

The relationship between adhesive capsulitis and diabetes mellitus (DM) is well-documented. The incidence of adhesive capsulitis is 2-4 times higher in diabetics than in the general population [14-15]. Patients with diabetes are at a higher risk of FS with an incidence of 10%-20% and patients having insulin dependence have an even higher incidence of 36% [16]. FS has been described as one of the most disabling musculoskeletal manifestations of DM. According to National Diabetes Information Clearinghouse (NDIC), the incidence of newly diagnosed diabetes cases aged 20 years or older was 1.3 million in the United States for the year 2005. The incidence of DM and the life expectancy of patients with diabetes have increased, resulting in an increase in the prevalence of musculoskeletal complications. The early diagnosis and effective management of DM reduce the risk of microvascular complications and the manifestations of organ involvement.

The prevalence of diabetes in patients with FS is not well addressed. This condition affects the ability to move the shoulder and usually has unilateral involvement. However, FS can also involve the other shoulder in approximately one of five patients [16]. FS is extremely uncommon among young people, and it is most commonly seen in individuals aged 40 to 60 years. According to a longitudinal study, female patients have a 1.6 times higher risk of developing this condition [17]. 

The pathophysiology of FS in patients with diabetes is still debatable. One theory involves collagen, a major building block of ligaments, tendons, and cartilage holding the bones together in a joint [18]. As more glucose molecules bind with collagen in people with diabetes, it leads to abnormal deposits of collagen in the cartilage and tendons of the shoulder [19-21]. This buildup then causes the affected shoulder to stiffen up. Inflammation and fibrosis also play a significant role in disease development [22]. The total prevalence of diabetes in patients with FS is 71.5% [23].

Overall, FS affects about 11%-30% of people with diabetes as compared to 2%-10% people without diabetes [24-25]. Patients who sustain a shoulder injury or undergo surgery of the shoulder can develop FS. When the injury is followed by prolonged joint immobilization, the risk of developing FS is the highest. A Southern California based study was conducted in 2007 to check the effect of glycemic control on FS. This retrospective study was based on the analysis of 201,513 patients with diabetes out of which 1150 had FS. It suggested no significant association between HbA1c levels and the prevalence of FS [25].

Gentle abduction, external rotation, and/or internal rotation at the level of the shoulder joint, within the limits of pain, has been recommended for long-term care in FS patients. Physiotherapy at this scale has a benefit, and most patients recover without complications [26-27]. For short-term symptom control, oral or injection steroid therapy is more effective and a remarkable improvement in motion can be achieved [28-29].

## Materials and methods

We designed a descriptive cross-sectional study and completed the research goals in 90 days in the months of April through June 2017. We conducted the research at the Diabetes Management Center, Services Hospital Lahore, Pakistan. It is a well-reputed medical center for the management of diabetes in the region. In our study, 2964 patients with diabetes from Lahore were registered. Patients coming from other areas of the province were excluded from the study. We also excluded patients less than 18 years of age and those with any history of shoulder or back trauma. After systematic randomization, we selected a sample of 80 patients. The patients agreed to participate, were explained the nature and objectives of this study, and informed consent was formally obtained. No reference to the patients' identity was made at any stage during data analysis or in the report. Our questionnaire included 10 disease-related questions in addition to the demographics of each patient. We collected the data by conducting questionnaire-based interviews. SPSS ver. 21.0 (IBM SPSS Inc, Chicago, IL, USA) was used for data analysis. Independent variables included age, sex, mode of anti-diabetic medication, exercise, the degree of glucose control, and the time since the diagnosis of diabetes. We analyzed the responses with dependent variables that included pain during movement of the shoulder, shoulder movement-restriction, stage of FS, and unilateral or bilateral pain/restriction. Diagnosis of FS was confirmed on radiologic findings. Results were analyzed using frequencies and crosstabs with the chi-square test to determine the significance of associations. An awareness program was also arranged for FS in this population at the auditorium of Services Hospital Lahore.

## Results

The sample population in our study consisted of patients with diabetes mainly of ages ranging from 41 to 60 years (56 of the 80 patients). The remaining patients were either older than 60 or were 18-40 years' old. There was a total of 42 female patients: 67.5% of the diabetic population under study had their diabetes diagnosed more than 5 years ago; 26 patients had their diabetes diagnosis made more than 10 years ago; and 37.5% of the population was taking insulin in some form with or without oral anti-diabetic medications. More than half of the population (53.8%) had their last blood-sugar levels in the normal glycemic range. The prevalence of FS was found out to be 41.3%, as 33 patients complained of pain during movement of the shoulder and had consistent radiologic findings. A total of 12 of them had bilateral shoulder involvement. Of those 33 patients, 16 faced restriction of movement below and above the shoulder while 17 reported only above-shoulder movement restriction. Of the 33, 20 had stage 1 FS while 13 had the stage 2 disease. Of the patients, 57.5% were doing regular cardiovascular exercises in the form of walking, jogging, cycling, swimming, or running, and 35% reported having an immediate family member (parents and/or siblings) who had a complaint of FS (Table 1).

**Table 1:** Demographics and response percentages of the sample population FS: frozen shoulder

Parameters		Percentage (%) of the total population (Count)
Age	18-40-year old	13.8 (11)
	41-60-year old	70.0 (56)
	61-80-year old	16.2 (13)
Gender	Males	47.5 (38)
	Females	52.5 (42)
Duration of diabetes	Less than 1 year	11.3 (9)
	1-5 years	21.3 (17)
	6-10 years	35.0 (28)
	More than 10 years	32.5 (26)
Mode of treatment	Oral	62.5 (50)
	Insulin	37.5 (30)
Glucose control	Controlled	53.8 (43)
	Uncontrolled	46.3 (37)
Shoulder pain	Present	41.3 (33)
	Absent	58.8 (47)
Location of pain	Unilateral	26.3 (21)
	Bilateral	15.0 (12)
Restriction of movement	Present	41.3 (33)
	Absent	58.8 (47)
Degree of restriction	Up to shoulder	20.0 (16)
	Above shoulder	21.3 (17)
Regular exercise	Practiced	57.5 (46)
	Not practiced	42.5 (34)
Family history	Present	43.8 (35)
	Absent	56.3 (45)
Stage of FS in diagnosed cases	Stage 1	25.0 (20)
	Stage 2	16.3 (13)
Total cases of FS diagnosed		41.3 (33)

On Pearson's chi-square analysis some interesting observations were made. The prevalence of FS was 2.66 times more in females than in males with x2 (9.216), df (1), p-value (0.002). Similarly, patients who were taking insulin with or without oral anti-diabetic medications were associated with a slightly higher frequency of FSx2 (6.963), df (1), p-value (0.008). Patients with uncontrolled blood glucose levels in the past 3 months also had a 1.5 times higher rate of developing shoulder pain: x2 (4.657), df (1), p-value (0.031). Those who had a family history of a similar ailment also had 1.5 times increased frequency: x2 (6.485), df (1), p-value (0.011). These associations were scrutinized and compared to reported findings in the previously published literature. Age distribution, duration of diabetes, and exercise had no significant relationship with the development of FS in our population (Table 2).

**Table 2:** Cross-analysis of probable determinants of FS with prevalence of the disease in the sample population using Pearson's chi-square test FS: frozen shoulder

Cross			Percentage (%) in group (Count)	x^2^	p-value
Age vs. FS	18-40-year old	Pain* present	12.1 (4)	2.500	.287
		Pain absent	14.9 (7)		
	41-60-year old	Pain present	78.8 (26)		
		Pain absent	63.8 (30)		
	61-80-year old	Pain present	9.1 (3)		
		Pain absent	21.3 (10)		
Sex vs. FS	Males	Pain present	27.3 (9)	9.216	.002
		Pain absent	61.7 (29)		
	Females	Pain present	72.7 (24)		
		Pain absent	38.3 (18)		
Duration of diabetes vs. FS	Less than 1 year	Pain present	3.0 (1)	7.287	.063
		Pain absent	17.0 (8)		
	1-5 years	Pain present	15.2 (5)		
		Pain absent	25.5 (12)		
	6-10 years	Pain present	36.4 (12)		
		Pain absent	34.0 (16)		
	More than 10 years	Pain present	45.5 (15)		
		Pain absent	23.4 (11)		
Mode of treatment vs. FS	Oral	Pain present	45.5 (15)	6.963	.008
		Pain absent	74.5 (35)		
	Insulin	Pain present	54.5 (18)		
		Pain absent	25.5 (12)		
Glucose control vs. FS	Controlled	Pain present	39.4 (13)	4.657	.031
		Pain absent	63.8 (30)		
	Uncontrolled	Pain present	60.6 (20)		
		Pain absent	36.2 (17)		
Regular exercise vs. FS	Practiced	Pain present	48.5 (16)	1.868	.172
		Pain absent	63.8 (30)		
	Not practiced	Pain present	51.5 (17)		
		Pain absent	36.2 (17)		
Family history vs. FS	Present	Pain present	60.6 (20)	6.485	.011
		Pain absent	31.9 (15)		
	Absent	Pain present	39.4 (13)		
		Pain absent	68.1 (32)		
Total diagnosed cases of FS			41.3 (33)		

*Note: Shoulder pain, especially during movement, was the main criterion used for the diagnosis of FS in these patients. All patients having shoulder pain also had restricted shoulder movements and were diagnosed with FS after ruling out other causes on radiologic analysis. Shoulder pain corresponds directly to the adhesive capsulitis in this data.

## Discussion

The present study is the first of its kind to estimate the prevalence of FS in a Pakistani population with diabetes. It also correlates FS with various demographic variables like age, gender, and nondemographic variables, such as glucose levels, time since onset of diabetes, mode of anti-diabetic medication, exercise, and family history.

Out of the 80 patients with diabetes evaluated, 41.3% had FS, while the international frequency is about 30% [24]. Among those 33 diabetic patients with shoulder pain, 100% had shoulder movement restriction; they were diagnosed with FS following consistent radiologic findings. In our study, FS was 2.66 times higher in female diabetics, which is higher than the internationally reported values [17,24]. A higher prevalence in this urban section of the Pakistani population, especially in women, can be attributed to poor socioeconomic status, late diagnosis, unawareness, lack of screening practices, poor glycemic control, and/or inadequate clinical management of FS.

Previously, studies have shown that the mode of anti-diabetic medications also affects the prevalence of FS. Insulin-dependent patients (whether using oral hypoglycemic agents or not) were 1.93 times more likely than non-insulin-dependent patients with diabetes to have FS, and that factor increased to 1.96 when the results were adjusted for HbA1c level [25]. Patients who were taking oral hypoglycemic drugs were 1.5 times more likely to develop FS than those who did not use insulin or oral hypoglycemic drugs [25]. However, the results of a recent meta-analysis showed no significant relationship between FS prevalence in patients with insulin-dependent diabetes mellitus or insulin-treated patients and that in non-insulin-dependent diabetes patients [26]. Our results showed 1.2 times higher rate of FS in patients on insulin with or without oral hypoglycemic agents. Sugar control showed a slightly significant association in the development of FS. In those with uncontrolled glucose levels in the past 3 months, 1.5 times higher rate of FS was observed (p=0.031). Hence, this association of glycemic control and FS needs to be substantiated with further studies.

In our study, 45.5% diabetic patients with shoulder pain were taking oral medications for glycemic control while the rest were taking insulin and the difference was statistically significant (p=0.008). Shoulder pain was unilateral in 21 (63.6%) patients, while 12 (36.4%) patients had bilateral involvement in the patient population in our study. It is reported in the literature that FS is mostly unilateral; however, 42% of the patients with bilateral FS had diabetes [26].

FS itself is not related to inheritance but its incidence in diabetic patients is higher and diabetes has a hereditary component in its multifactorial inheritance. Some authors have suggested a link between genetic factors and the etiology of FS but others could not confirm such observations. An increased frequency of human leukocyte antigen B27 (HLA-B27) in patients with FS was reported but this association has also not been validated to date [27]. The present study shows that FS can have a significant hereditary association, as 20 out of 33 patients had a positive family history.

Exercise has no effect on prevalence but has some role in the management of FS. Although the patients who were treated with exercise techniques regularly had a slightly better prognosis, no significant association was seen with regular exercise [28]. In patients with a relatively acute disease, oral or injection steroid therapy is more effective and a remarkable improvement in motion can be achieved. Therefore, steroid therapy can be employed on a short-term basis [29-30].

Due to the smaller sample size, our study has its limitations. We could not find a significant association in our results with the duration of diabetes. Previous studies have shown that the duration of diabetes is related to the development of FS after controlling for insulin use (odds ratio: 1.85 for the duration of more than 10 years of use compared to those with less than 5 years of use) [25]. The prevalence of end-stage diabetic manifestations increased in patients with FS as compared to those without FS (p < 0.0001) [25]. It can be attributed to a possible component of recall bias and/or insufficient sample. Similarly, a lack of association with age was also found in our results. Therefore, further population-based studies are required to be conducted in Pakistan to validate these results.

## Conclusions

Effective glycemic control and early FS management can promise a higher level of productivity in patients with diabetes. Awareness programs should be launched targeting the populations with diabetes, especially women, at regular intervals to provide information about the increasing prevalence, symptoms, and risk factors of FS. Initial screening and shoulder radiographs should be performed for patients with diabetes with suspected FS as early detection favors better cure and late detection worsens clinical outcomes. Moreover, physicians should update their clinical knowledge regarding this association as it holds paramount importance in the diagnosis and management of FS in patients with diabetes. Larger, multicenter studies are needed to investigate FS further in these populations.
